# Size dependence- and induced transformations- of fractional quantum Hall effects under tilted magnetic fields

**DOI:** 10.1038/s41598-022-22812-x

**Published:** 2022-11-10

**Authors:** U. Kushan Wijewardena, Tharanga R. Nanayakkara, Annika Kriisa, Christian Reichl, Werner Wegscheider, Ramesh G. Mani

**Affiliations:** 1 Georgia State University, Atlanta, GA 30303 USA; 2 ETH-Zurich, 8093 Zurich, Switzerland

**Keywords:** Condensed-matter physics, Condensed-matter physics

## Abstract

Two-dimensional electron systems subjected to high transverse magnetic fields can exhibit Fractional Quantum Hall Effects (FQHE). In the GaAs/AlGaAs 2D electron system, a double degeneracy of Landau levels due to electron-spin, is removed by a small Zeeman spin splitting, $g \mu _B B$, comparable to the correlation energy. Then, a change of the Zeeman splitting relative to the correlation energy can lead to a re-ordering between spin polarized, partially polarized, and unpolarized many body ground states at a constant filling factor. We show here that tuning the spin energy can produce fractionally quantized Hall effect transitions that include both a change in $\nu$ for the $R_{xx}$ minimum, e.g., from $\nu = 11/7$ to $\nu = 8/5$, and a corresponding change in the $R_{xy}$, e.g., from $R_{xy}/R_{K} = (11/7)^{-1}$ to $R_{xy}/R_{K} = (8/5)^{-1}$, with increasing tilt angle. Further, we exhibit a striking size dependence in the tilt angle interval for the vanishing of the $\nu = 4/3$ and $\nu = 7/5$ resistance minima, including “avoided crossing” type lineshape characteristics, and observable shifts of $R_{xy}$ at the $R_{xx}$ minima- the latter occurring for $\nu = 4/3, 7/5$ and the 10/7. The results demonstrate both size dependence and the possibility, not just of competition between different spin polarized states at the same $\nu$ and $R_{xy}$, but also the tilt- or Zeeman-energy-dependent- crossover between distinct FQHE associated with different Hall resistances.

## Introduction

Two-dimensional electron systems subjected to high transverse magnetic fields can exhibit Fractional Quantum Hall Effects (FQHE), which signify incompressible correlated electronic states in the vicinity of mostly odd- and some even-denominator rational fractional filling factors, $\nu \sim p/q$, of Landau levels^1–3^. Although graphene^4–6^ has recently become an interesting material for studying FQHE^7–13^, along with the ZnO based system^14–16^, the GaAs/AlGaAs system, due to its extra-ordinarily high quality, is still a material of choice for studying related phenomena^3,17,18^. In the GaAs/AlGaAs 2D electron system, a double degeneracy of Landau levels due to electron-spin, is removed by a small Zeeman spin splitting, $g \mu _B B$, comparable to the correlation energy. Then, a change of the Zeeman splitting relative to the correlation energy can lead to a re-ordering between spin polarized, partially polarized, and unpolarized many body ground states at a constant filling factor^19–24^. Halperin identified the possibility of a spin-unpolarized FQHE ground state having a lower energy than the spin polarized ground state at, e.g., $\nu = 2/5$, for a system exhibiting a small spin splitting^19^. Thus arose searches for induced-transitions between unpolarized and polarized ground states, upon changing the Zeeman energy, at a constant $\nu$. An experimentally observed transition, characterized by a sharp change in the angular dependence of the activation energy, between two distinct fractional quantum Hall states at a constant $\nu = 8/5$, was attributed to a transition from a spin-unpolarized state to a spin polarized state with increased angle^21^. Another study used tilt field measurements to infer a polarized 5/3 state, and a field induced unpolarized to partially polarized transition at a constant $\nu = 4/3$^22^. Eisenstein et al. also reported a re-entrant 2/3 energy gap from activation studies, suggestive again of a transition from a spin unpolarized to spin polarized state with increasing total magnetic fields^23^. See also^25^. Engel et al., reported a tilt angle dependent splitting of the 2/3 and 3/5 states associated with a spin transition^24^. In all these above-mentioned studies, examined state transitions occur at a constant filling factor. Since such state transitions occur about a constant filling factor ($\nu \sim p/q$), the initial and final states have the same quantized Hall resistance, i.e., $R_{xy} = (p/q)^{-1}(h/e^{2})$. More recently, Feldman et al. examined the compressibility in suspended exfoliated graphene, and reported phase transitions marked by regions of negative compressibility that cut across incompressible peaks at FQHE filling factors^9^. Since standard $R_{xx}$ and $R_{xy}$ measurements were not reported, it is not known whether or not different phases correspond to the same Hall resistance. Other related studies include^32–36^. In this work, we show that tilting a specimen to change the spin energy can produce fractionally quantized Hall effect transitions that include both a change in $\nu$ for the $R_{xx}$ minimum, e.g., from $\nu = 11/7$ to $\nu = 8/5$, and a corresponding change in the $R_{xy}$, e.g., from $R_{xy}/R_{K} = (11/7)^{-1}$ to $R_{xy}/R_{K} = (8/5)^{-1}$, with increasing tilt angle. Further, we exhibit a striking size dependence in the tilt angle interval for the vanishing of the $\nu = 4/3$ and $\nu = 7/5$ resistance minima, including “avoided crossing” type lineshape characteristics, and observable shifts of $R_{xy}$ at the $R_{xx}$ minima- the latter occurring for $\nu = 4/3, 7/5$ and the 10/7. The results demonstrate both size dependence and the possibility, not just of competition between different spin polarized states at the same $\nu$ and $R_{xy}$, but also the tilt- or Zeeman-energy-dependent- crossover between distinct FQHE associated with different Hall resistances.

## Results

**Figure 1:**
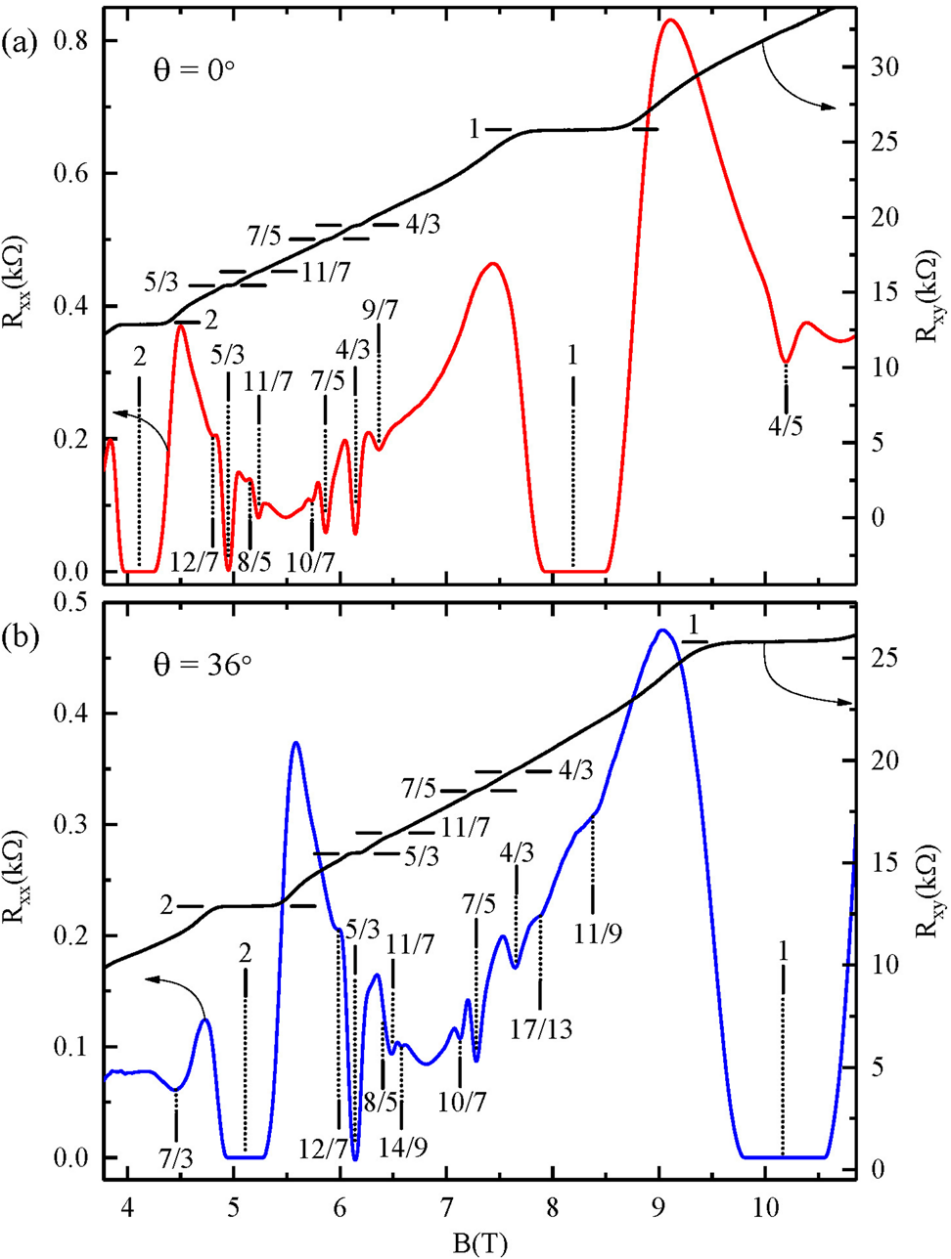
The diagonal ($R_{xx}$) and Hall ($R_{xy}$) resistances are exhibited for a GaAs/AlGaAs heterostructure device at tilt angles $\theta = 0^{0}$ and at $\theta =36^{0}$. $R_{xx}$ and $R_{xy}$ are shown for magnetic fields $3.7<B<10.8$ Tesla at $T=55\,mK$, highlighting integral- and fractional- quantum Hall effects, with the magnetic field oriented perpendicular to the 2DES, i.e., $\theta = 0^{0}$ in panel (**a**), and with the 2DES tilted by $\theta = 36^{0}$ with respect to the magnetic field in panel (**b**). Here, the device width $W = 400\, \mu m$.

A defining characteristic of FQHE is a simple inverse relation between the filling factor ($\nu \sim p/q$) for the $R_{xx}$ minimum and the normalized plateau Hall resistance $R_{xy}/R_{K} = (p/q)^{-1}$, where $R_{K} = 25.812 k \Omega$^1,2^. At $\nu \le 1$, FQHE occur prominently, about $\nu = p / (2kp \pm 1)$, on either side of the half-filled ($\nu = 1/2$) state^26–28^. In the GaAs/AlGaAs 2D electron system, FQHE become manifested also in the upper spin Landau subband about $\nu = 1+ p / (2kp \pm 1)$, for example, about $\nu = 4/3, 7/5, 10/7, ...$ for $\nu \le 3/2$ and about $\nu =2,5/3, 8/5, 11/7...$ for $\nu \ge 3/2$^29,30^. Figure 1 exhibits such FQHE in the $R_{xx}$ and $R_{xy}$ traces versus the magnetic field, *B*, with tilt angle $\theta = 0^{0}$, (see Fig. 1a), and with the 2DES-normal rotated by $\theta = 36^{0}$ (Fig. 1b) with respect to the *B*-axis, where the *B*-interval includes $1 \le \nu \le 2$, the topic of this study, for a $W=400 \,\mu m$ Hall bar device. This device, fabricated from GaAs/AlGaAs single heterostructures, was characterized by a sheet electron density $n_{0} (55mK) = 2 \times 10^{11} cm^{-2}$ and an electron mobility $\mu (55\,mK) = 1.4 \times 10^{7} \,cm^2/Vs$ after brief illumination.The thickness of the 2D electron system is estimated to be ca. 50 nm. For $\nu \le 3/2$, Fig. 1a exhibits FQHE features at 4/5, 9/7, 4/3, 7/5, and 10/7, while for $\nu \ge 3/2$, FQHE features are observable at 5/3, and 11/7. At $\theta =36^{0}$, for $\nu \le 3/2$, Fig. 1b exhibits FQHE features at 11/9 instead of 9/7, plus 17/13 which is not visible in Fig. 1a, in addition to 4/3, 7/5, and 10/7. In Fig. 1b, for $\nu \ge 3/2$, FQHE features are observable at 5/3, and 11/7 as in Fig. 1a. In addition, 7/3 is visible, with a weak feature at 14/9. Note also that, a resistance minimum is not observable at 8/5 in both Fig. 1a and b, while QHE at 1 and 2 are observable in both panels. The oscillatory pattern is shifted to higher *B* in Fig. 1b with respect to Fig. 1a because Landau quantization depends on $B_{\perp }$, while $B = B_{\perp }/cos(\theta )$ is plotted on the abscissa in Fig. 1, and experiment is limited to $0 < cos(\theta ) \le 1$ for $90^{0} > \theta \ge 0^{0}$.

**Figure 2:**
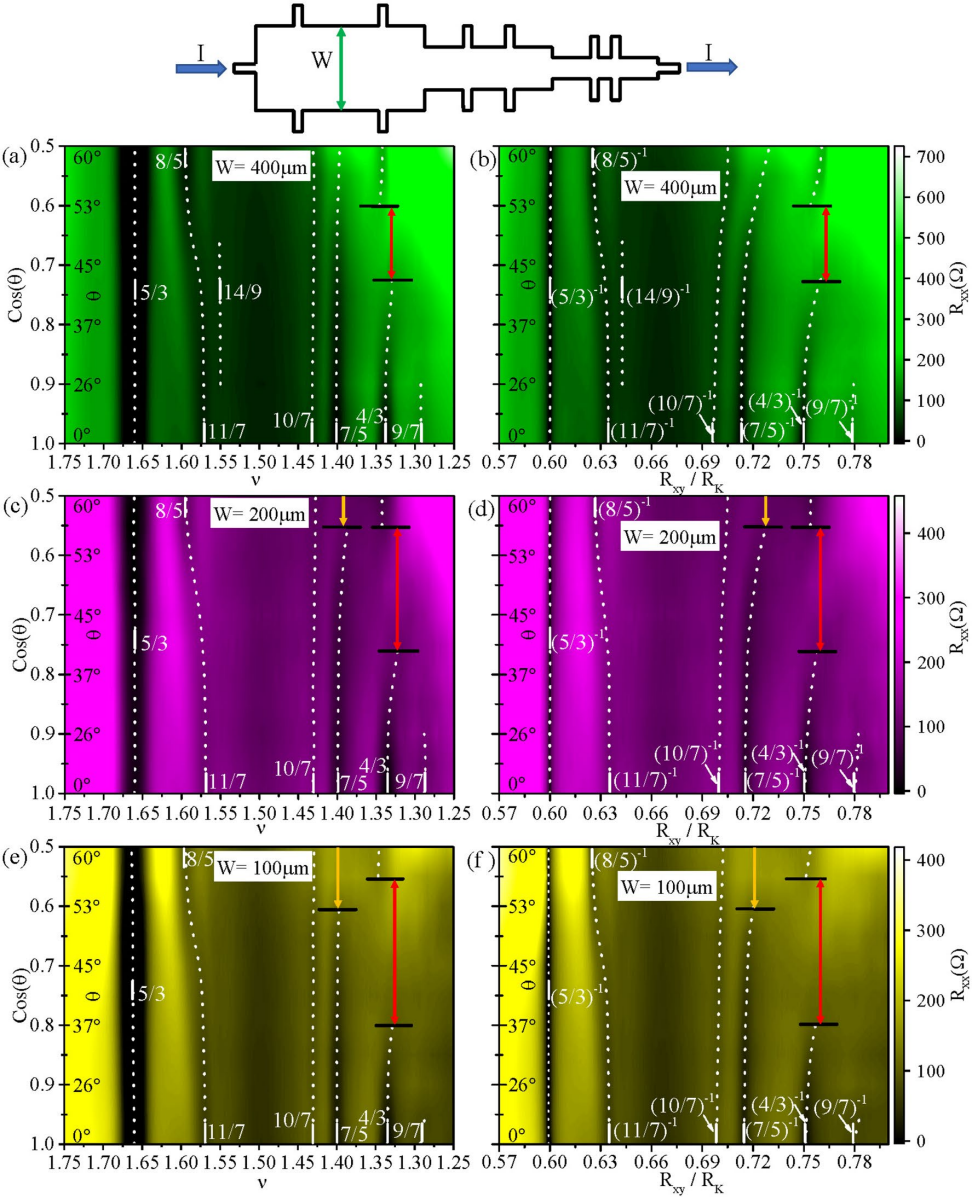
Color plots of the tilt field effect in Hall bars with different widths (*W*). (Top): The Hall bar geometry including three different widths, *W*, with length (L) to *W* ratio for the contacted regions L/W = 1. (**a**), (**c**) and (**e**) depict color plots of $R_{xx}$ versus cos($\theta$) and versus $\nu$ for *W*= 400, 200, and 100 $\mu m$, respectively. (**b**), (**d**) and (**f**) shows color plots of $R_{xx}$ versus cos($\theta$) and versus $R_{xy}/R_{K}$ for *W*= 400, 200, and 100 $\mu m$, respectively. The dotted lines follow the $R_{xx}$ minima. The horizontal lines in black, with the colored vertical arrowed lines, mark the boundary of the size-dependent angular interval where the diagonal resistance minimum vanishes. Here, $T=$ 55 mK.

Such traces of the $R_{xx}$ and $R_{xy}$ were obtained at angles $\theta$ selected to realize equal increments in $cos(\theta )$, and the measurements were carried out simultaneously in a Hall device including three sections with width $W = 400, 200$ and $100\, \mu m$, see Fig. 2(top)^31^. The applied *B* was transformed to $B_{\perp } = B cos (\theta )$, and then to $\nu = hn/eB_{\perp }$, where *n* is the electron density, *h* is Planck’s constant, and *e* is the electron charge. The resulting line traces were compiled into color plots, as shown in Fig. 2a, c and e. Here, the abscissas show $\nu$. Dark bands in the figure indicate resistance minima; short white vertical bars within the figure panels indicate $\nu$ associated with preeminent FQHE in the exhibited $\nu$ interval. In Fig. 2a, c and e, the 5/3 FQHE minimum is prominent and it runs vertically. In sharp contrast, the 11/7 $R_{xx}$ minimum, which is prominent at $\theta =0^{0}$ in Fig. 1a, and visible here in Fig. 2a, c and e in the vicinity of $cos(\theta )=1$ ($\theta = 0^{0}$), deviates towards higher $\nu$ (lower $B_{\perp }$) with decreasing $cos(\theta )$ (higher angle), and this resistance minimum ends up at $\nu = 8/5$ at $cos(\theta ) = 0.65$ ($\theta \sim 50 ^{0}$). A $R_{xx}$ minimum at 14/9 occurs over a short angular interval only in Fig. 2a. The 10/7 and the 7/5 FQHE minima run mostly vertically in the versus $\nu$ plots of Fig. 2a and e. The 7/5 minimum appears to terminate at a smaller angle in the smaller *W* sections. For example, the 7/5 terminates at $\theta < 53^{0}$ in $W =100\, \mu m$ section, at $\theta = 56^{0}$ for $W = 200 \,\mu m$, while it decays towards $\theta = 60^{0}$ in the $W = 400 \,\mu m$ section.

In Fig. 2a, c and e, the 4/3 is also prominent near $cos(\theta ) = 1$, then with increasing angle, it vanishes at around $cos(\theta ) = 0.73$ in $W= 400 \,\mu m$ and around $cos(\theta ) = 0.81$ in $W=100 \,\mu m$, and then reappears at smaller $cos(\theta )$ (larger $\theta$). A striking feature, which is indicated by the black horizontal lines for the 4/3, is an apparent size dependence in the span of angles over which the 4/3 state vanishes (cf. Fig. 2a–c). The figures indicate that the 4/3 vanishes over a broader range of angles in the narrower specimen. Thus, for example, in the $W = 100 \,\mu m$ specimen, the 4/3 resistance minimum vanishes between $36^{0} \le \theta \le 56^{0}$, for the $W = 200 \,\mu m$ specimen, it vanishes between, $41^{0} \le \theta \le 56^{0}$, while for the $W = 400\, \mu m$ specimen the same occurs over the narrower interval $43^{0} \le \theta \le 53^{0}$. Finally, there is also a minimum at 9/7 that is only visible for $cos(\theta ) > 0.92$.

The color plots of Fig. 2a, c and e convey the behavior of the $R_{xx}$ versus the tilt angle and the filling factor $\nu$. In order to track the correlation between $R_{xx}$ and $R_{xy}$, color plots of $R_{xx}$, with $R_{xy}/R_{K}$ as the abscissa, and $cos(\theta )$ as the ordinate are exhibited in Fig. 2b, d and f. Fig. 2b, d and f show some of the same general features as Fig. 2a, c and e including these differences: (i) The 5/3 minimum, for example, is narrower in Fig. 2b, d and f in comparison to Fig. 2a, c and e. This feature follows from the flattening of the Hall resistance versus *B* about FQHE $R_{xx}$ minima. As $R_{xx}$ occurring at different *B* then tend to show the same $R_{xy}$ over the $R_{xx}$ minimum, there occurs a “compression” of the $R_{xx}$ minima about the Hall plateau value, which is what is seen in Fig. 2b, d and f. (ii) Fig. 2b, d and f confirm that, as the $\nu = 11/7$ $R_{xx}$ minimum moves in filling factor to $\nu =8/5$ in Fig. 2a, c and e, the associated $R_{xy}$ shifts from $R_{xy}/R_{K}= (11/7)^{-1}$ to $R_{xy}/R_{K}= (8/5)^{-1}$. (iii) While Fig. 2a, c and e suggest a disappearance and reentrance of the $R_{xx}$ minimum in the vicinity $\nu = 4/3$ with decreasing $\cos (\theta )$, Fig. 2b, d and f suggest also a corresponding shift in the $R_{xy}/R_{K}$ such that, about “re-entrance”, the $R_{xy}/R_{K}$ differs perceptibly from $R_{xy}/R_{K} = (4/3)^{-1}$. That is, at the largest angles, the “$\nu = 4/3$” state exhibits a different $R_{xy}/R_{K}$ relative to $R_{xy}/R_{K} = (4/3)^{-1}$ observed at the smallest angles. (iv) An “avoided crossing” type trajectory for 4/3 about its re-entrance in Fig. 2a, c and e appears even more pronounced in the Fig. 2b, d and f. Again, the angular span over which this $R_{xx}$ minimum disappears depends on the specimen width *W*, with disappearance over larger angular span at the smaller *W*. (v) The $\nu = 7/5$ state, which shows a mostly vertical trajectory in Fig. 2a and e, shows strong bending to larger $R_{xy}/R_{K}$ at large angles in Fig. 2b, d and f indicating a deviation from $R_{xy}/R_{K} = (7/5)^{-1}$. (vi) The 10/7 also shows curvature in $R_{xy}/R_{K}$ towards higher $R_{xy}/R_{K}$ values in all three sections.

**Figure 3:**
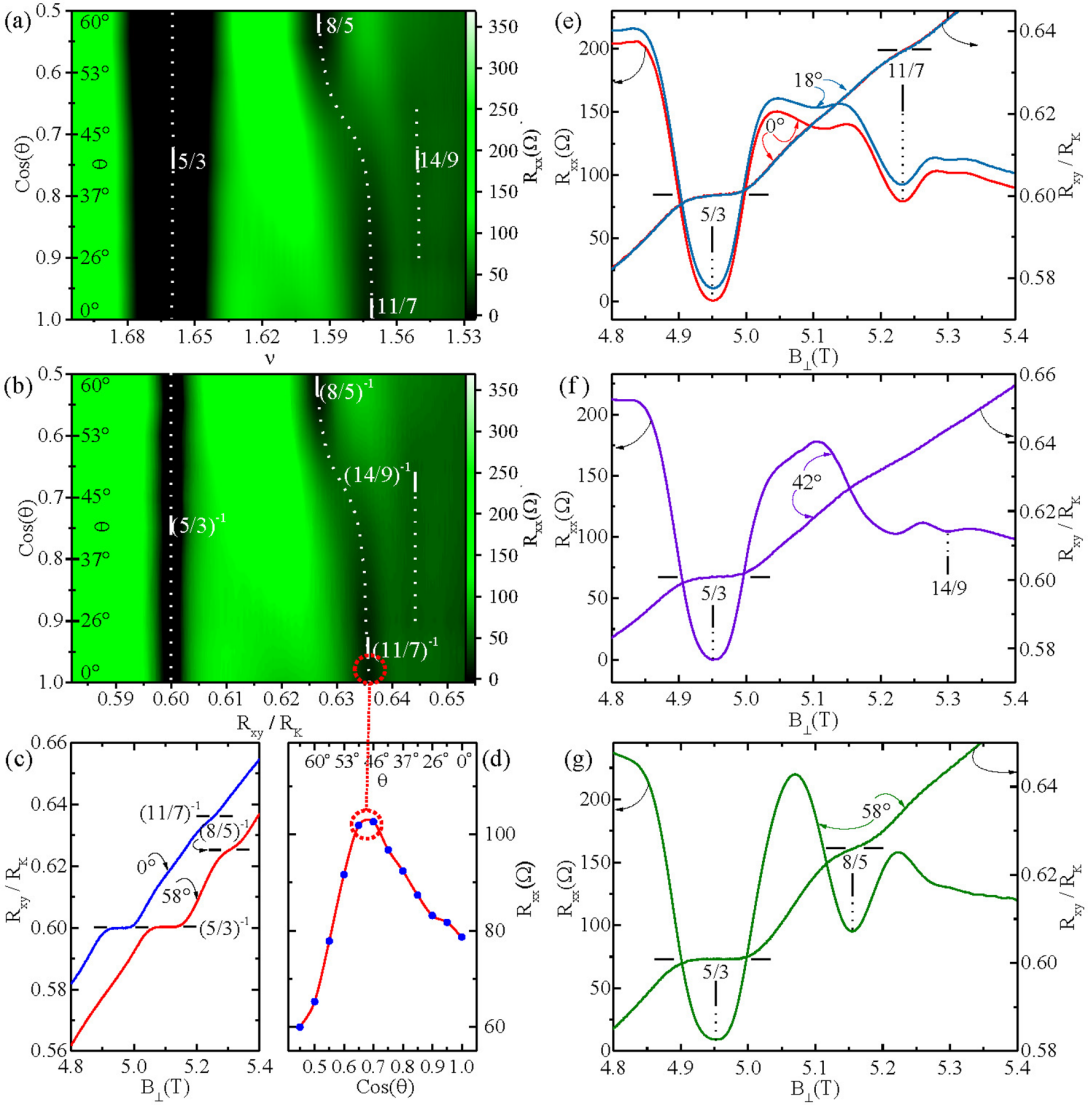
Detailed view of ’11/7’ to ’8/5’ transformation with tilt angle ($\theta$) for $W=400\, \mu m$. (**a**) A color plot of $R_{xx}$ versus cos($\theta$) (ordinate) and versus $\nu$ (abscissa). (**b**) A color plot of $R_{xx}$ versus cos($\theta$) (ordinate) and versus $R_{xy}/R_{K}$ (abscissa). The dotted lines indicate the trajectories of $R_{xx}$ minima. The color bars on the right in (**a**) and (**b**) indicate the magnitude of $R_{xx}$. (**c**) $R_{xy}/R_{K}$ is plotted against $B_{\perp }$ at $\theta =0^{0}$ and $\theta = 58^{0}$. The traces have been offset along the abscissa by 0.15 Tesla, for the sake of clarity. (**d**) This panel shows $R_{xx}$ along the resistance minimum [dotted line in (**b**)] that connects the ’11/7’ and ’8/5’ FQHE as a function of ($\theta$). (**e**), (**f**), and (**g**) depict the $R_{xy}/R_{K}$ and $R_{xx}$ traces plotted against $B_{\perp }$ at $\theta =( 0^{0}, 18^{0}), 42^{0},$ and $58^{0}$, respectively. The $R_{xx}$ trace at $18^{0}$ in (**e**) is offset along the ordinate by $10\, \Omega$.

Figure 3 highlights the tilt-field induced crossover between the 11/7 and the 8/5 in the $W=400 \,\mu m$ section. Figure 3a and b show color plots of $R_{xx}$ with $cos(\theta )$ on the ordinate as Fig. 3a shows $\nu$ as the abscissa, while Fig. 3b shows $R_{xy}/R_{K}$ on the abscissa. Figure 3b shows the narrowing of the 5/3 dark-band resistance-minimum in the $R_{xy}/R_{K}$ plot, as noted. The remarkable feature highlighted in this color plot is the sharp tilt-induced-crossover from a 11/7 FQHE to the 8/5 FQHE over the narrow angular interval $41^{0} \le \theta \le 49^{0}$. Figure 3c shows line traces of the Hall resistance at two extremal angles. At $\theta = 0^{0}$, a Hall plateau does not occur at 8/5, as this plateau becomes visible at $\theta = 58^{0}$. Figure 3d shows the $R_{xx}$ along the $11/7 \rightarrow 8/5$ resistance valley or minimum (dotted line) as a function of $\theta$ (top) and $cos(\theta )$ (bottom). The figure indicates that $R_{xx}$ initially increases with $\theta$ before decreasing for $\theta \ge 48^{0}$. Figure 3e–g exhibit some representative line traces of $R_{xx}$ and $R_{xy}$ that were used to build up the color plots shown in Fig. 3a and b.

**Figure 4:**
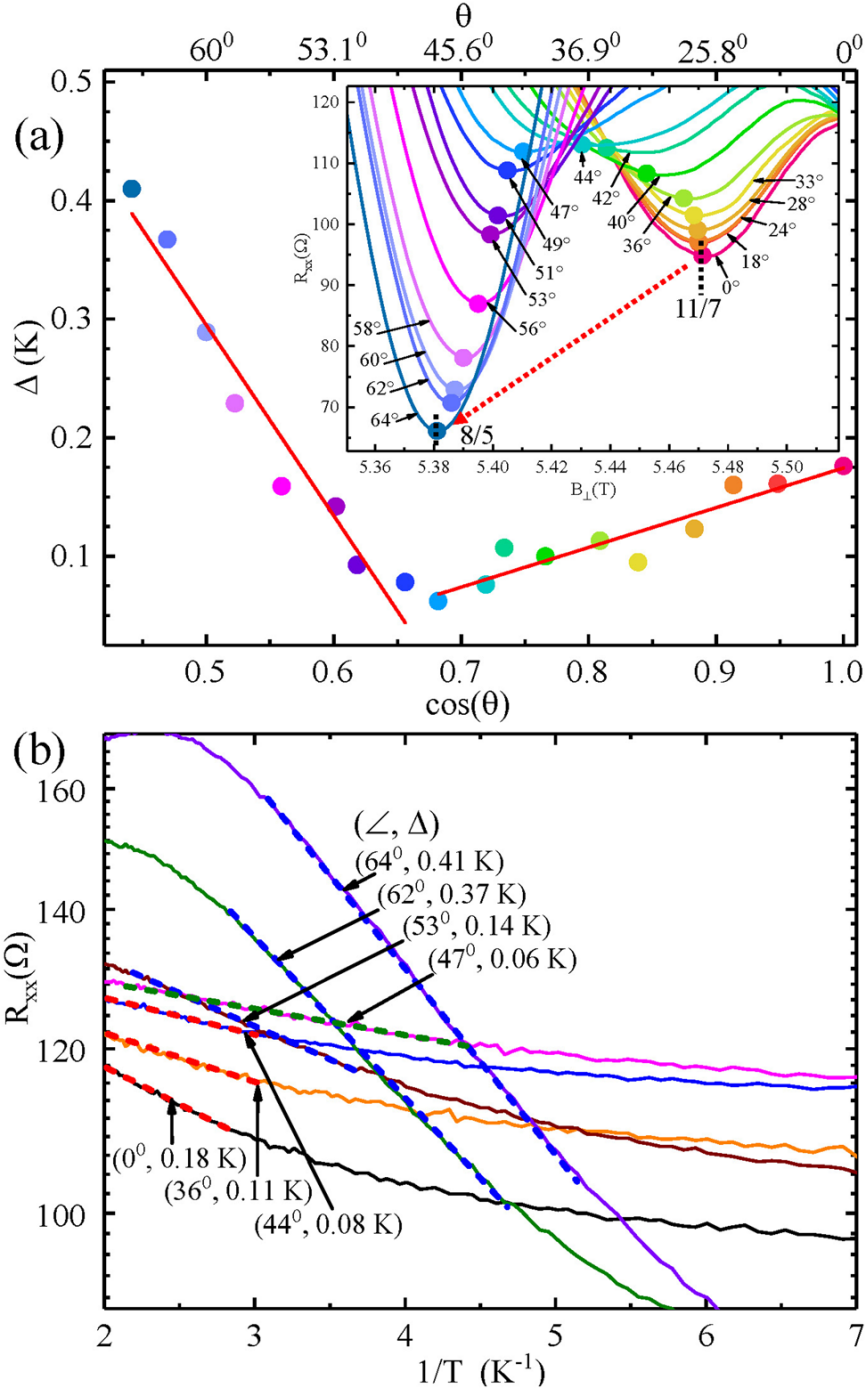
Activation energies in the ’11/7’ to ’8/5’ transformation with tilt angle ($\theta$) (**a**) The activation energy $\Delta$ is plotted vs cos($\theta$) and $\theta$, where $\theta$ is the tilt angle, for $W=400\, \mu m$. The figure shows that the $\Delta$ decreases with increasing angle until $\theta \approx 47^{0}$, before beginning to increase with $\theta$. The inset shows the $R_{xx} vs. B_{\bot}$ traces highlighting the $11/7-8/5$ crossover versus $\theta$ at base temperature. (**b**) $R_{xx}$ versus 1/*T* traces are exhibited for various angles $\theta$ here along with extracted activation energies, which are plotted in (**a**).

Figure 4 summarizes measurements of the activation energies $\Delta$ as a function of $cos(\theta)$ over the observed $11/7 \rightarrow 8/5$ crossover, while the inset shows the $R_{xx}$ vs $B_{\perp }$ at the various tilt angles $\theta$. As in Fig. 3d, the Fig. 4 (inset) shows that $R_{xx}$ at the minima initially increase with increasing $\theta$ before decreasing for $\theta \ge 47^{0}$. Figure 4 shows that the $\Delta$ decrease initially with increasing $\theta$ before increasing once again for $\theta \ge 47^{0}$. These results (Figs. 2, 3, 4) indicate that tilting the specimen can not only induce a crossover from one spin polarized state to another at the same filling factor as previously understood, but that tilt can also induce a crossover from one FQHE to another distinct FQHE.

**Figure 5:**
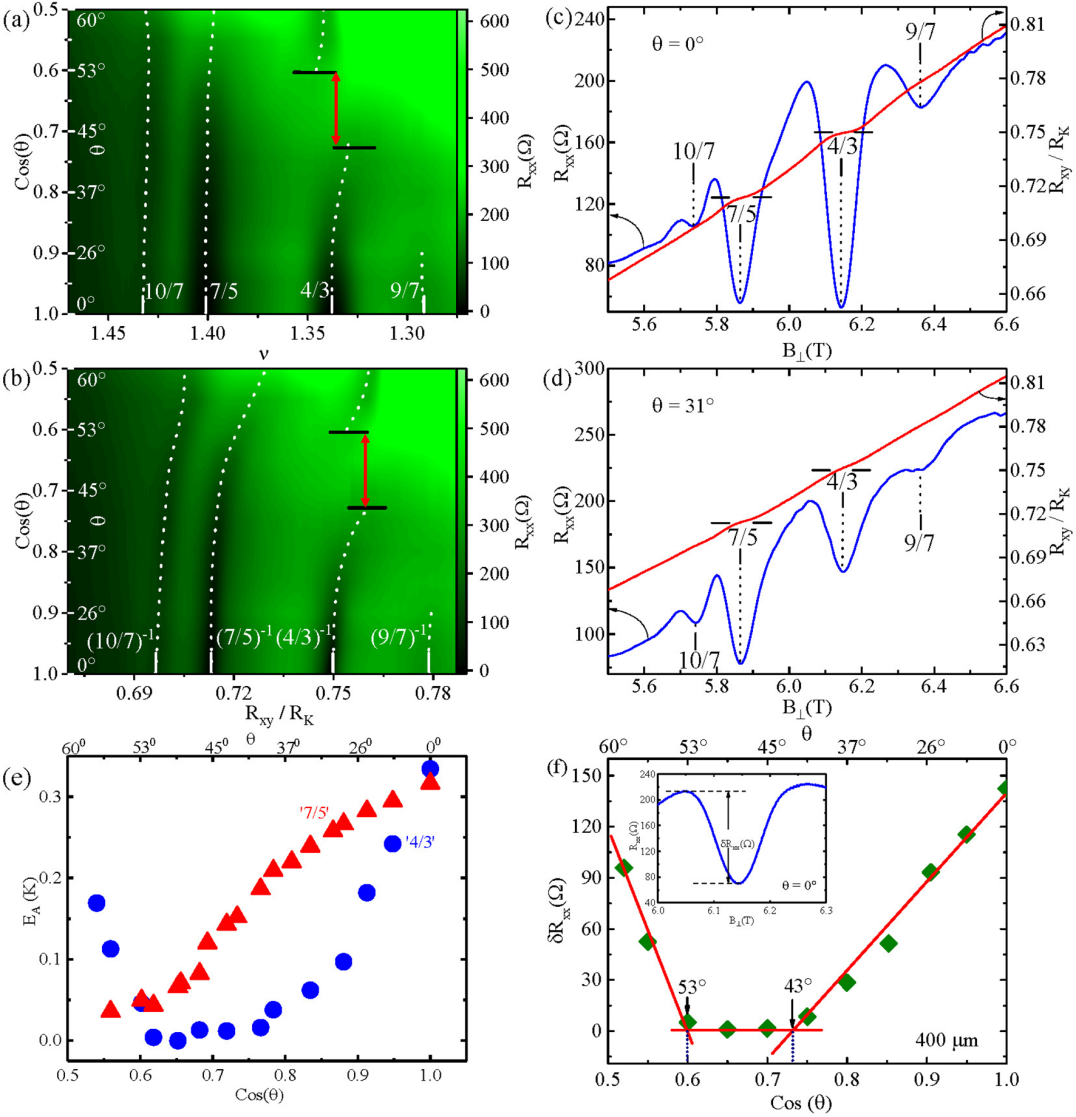
The trajectory of the resistance minima versus tilt angle for $\nu \le 3/2$ and $W= 400\, \mu m$. (**a**) A color plot of $R_{xx}$ versus cos($\theta$) and versus $\nu$. (**b**) A color plot of $R_{xx}$ versus cos($\theta$) and versus $R_{xy}/R_{K}$. Note the disappearance and re-entrance of FQHE near $\nu = 4/3$ along with a tilt angle dependent shift away in (**b**) from $R_{xy}/R_{K} = (4/3)^{-1}$ with increasing $cos(\theta ).$ Here, the arrowed red vertical lines mark the boundary of the angular interval where the $R_{xx}$ minima vanish. Note the $cos(\theta )$ dependent shifts also for 10/7 and 7/5. (**c**) and (**d**) show the $R_{xy}/R_{K}$ and $R_{xx}$ traces plotted against $B_{\perp }$ at $\theta = 0^{0}$ and $31^{0}$, respectively. (**e**) The activation energies, $E_{A}$, as a function of $cos(\theta )$ for the 4/3 and 7/5, respectively. Note that the $E_{A}$ tends to vanish over angles where the resistance minima vanish. (**f**) The angular span where $R_{xx}$ vanishes is determined by plotting $\delta R_{xx}$, see inset, versus $cos(\theta )$, as shown for 4/3.

Figure 5 highlights the observed resistance minima at $\nu \le 3/2$ for $W = 400\, \mu m$. Figure 5a and b show color plots of $R_{xx}$ with $cos(\theta )$ on the ordinate, as Fig. 5a shows $\nu$ as the abscissa, while Fig. 5b shows $R_{xy}/R_{K}$ on the abscissa. In Fig. 5a, we note that the $R_{xx}$ minimum around $\nu = 4/3$, appears very deep at $\theta = 0^{0}$, see also Fig. 5c, but then becomes weaker with increasing $\theta$, see also Fig. 5d, before vanishing entirely by $\theta = 43^{0}$ (Fig. 5a). The resistance minimum reappears at $\theta = 53^{0}$. Fig. 5b, which includes $R_{xy}/R_{K}$ as the abscissa, shows up to a $\approx 2 \%$ shift in the Hall resistance after re-entrance to a value above $R_{xy}/R_{K} = 0.75 = (4/3)^{-1}$. Note that the shape of the dotted lines associated with the “4/3” in Fig. 5b are reminiscent of “avoided crossing” type lineshape characteristics. Figure 5b also indicates shifts in the 7/5 and 10/7 towards higher Hall resistances with increasing tilt angles. These curved dotted lines associated with the 7/5 and the 10/7 in Fig. 5b suggest incomplete tilt-induced transitions which may, perhaps, require higher tilt angles and higher fields, beyond what is possible in our setup, to complete the transition. Figure 5e exhibits the activation energies as a function of $cos(\theta )$ (lower abscissa) for the 4/3 and 7/5 resistance minima. Figure 5e shows that the $E_{A}$ tends to vanish over the angular interval where the 4/3 minima vanish in Fig. 5a and b. Figure 5e also shows that for the 7/5, $E_{A} \rightarrow 0$ as $\theta \rightarrow 60^{0}$. Figure 5f illustrates the method utilized to determine the angular span over which the resistance minima vanish. Here, we measured the depth ($\delta R_{xx}$) of the $R_{xx}$ minimum as shown in the inset of Fig. 5f, plotted the $\delta R_{xx}$ versus $cos(\theta )$, approximated the wings by straight lines, to determine the angular boundaries, as illustrated in Fig. 5f.

**Figure 6:**
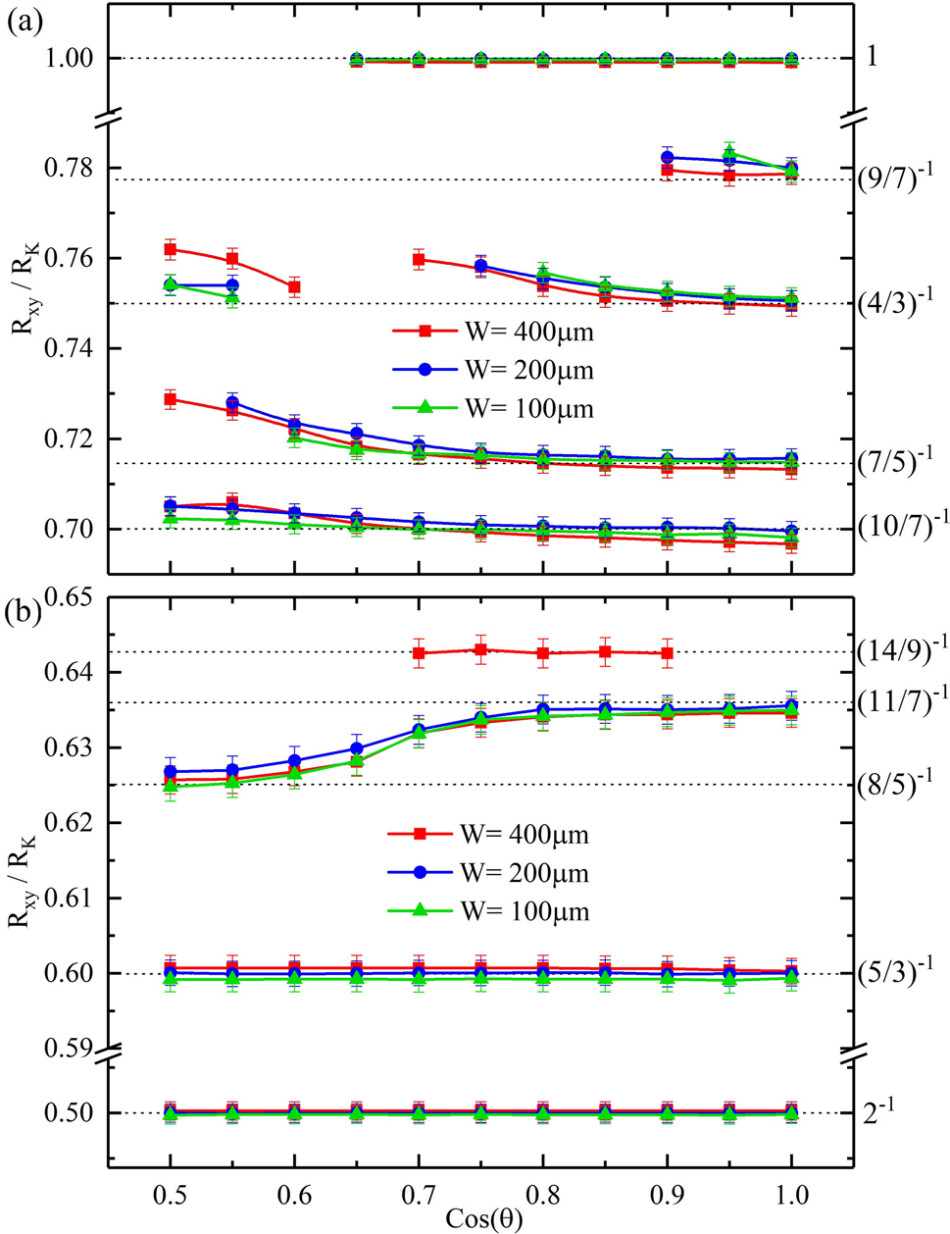
Measured $R_{xy}/R_{K}$ values at the diagonal resistance minima versus cos($\theta$) in Hall bars with $W = 400, 200,$ and $100\, \mu m$. (**a**) and (**b**) illustrate the observed $R_{xy}/R_{K}$ versus cos($\theta$), where $\theta$ is the tilt angle, at the corresponding $R_{xx}$ minima for the sample widths of 400, 200, and 100 micrometers. The dotted lines indicate the expected $R_{xy}/R_{K}$ values at the well known fractional states labeled on the right ordinate. Note that, at $\nu =p/q$ , where *p*/*q* is a rational fraction, one expects Hall resistance $R_{xy}/R_{K} = (p/q)^{-1}$.

Figure 6 summarizes the Hall resistances obtained at the $R_{xx}$ minima as a function of $cos(\theta )$ for $\nu \le 3/2$ in Fig. 6a, and $\nu \ge 3/2$ in Fig. 6b, for $W = 400, 200,$ and $100\,\mu m$. Figure 6a shows, unsurprisingly, that for $\nu = 1$, the $R_{xy}/R_{K} = 1$, for the entire examined range of $\theta$. The 4/3 state exhibits the proper value $R_{xy}/R_{K} = 0.75 = (4/3)^{-1}$ in the absence of tilt within experimental uncertainties. Increasing the $\theta$, or equivalently, decreasing $cos(\theta )$, produces a progressive shift in $R_{xy}/R_{K}$ towards higher values in specimens of all three sizes, until the 4/3 state disappears. When it re-appears near $cos(\theta ) = 0.6$ for $W=400 \, \mu m$, remarkably, the $R_{xy}/R_{K}$ appears shifted downwards relative to the last observable $R_{xy}/R_{K}$ value before disappearance. Then, it begins to increase once again with decreasing $cos(\theta )$. Note that the span of angles, where the 4/3 state vanishes, depends also on the size of the device, with the largest span for the vanishing minima in the narrowest specimen. The 7/5 and the 10/7 also indicate a progressive shift to larger $R_{xy}/R_{K}$ with decreasing $cos(\theta )$ or increasing $\theta$. Figure 6b shows that the $R_{xy}/R_{K} =0.5 = 2^{-1}$ for the $\nu = 2$ QHE and $R_{xy}/R_{K} = 0.6 = (5/3)^{-1}$ for the $\nu = 5/3$ FQHE, independent of the tilt angle or $cos(\theta )$. On the other hand, the 11/7 FQHE turns into the 8/5 FQHE with increasing tilt angle or decreasing $cos(\theta )$. The 14/9 FQHE, which makes a brief appearance for an intermediate set of angles in the $W = 400 \,\mu m$ section, shows the expected $R_{xy}/R_{K}$ within experimental uncertainties.

## Discussion

Here, we experimentally examined the diagonal and Hall resistances, observe dissimilar dependence when the results are plotted vs $\nu$ (Fig. 2a, c and e), and versus $R_{xy}/R_{K}$ (Fig. 2b, d and f), respectively, and report a striking tilt-induced crossover from one fractional quantized Hall resistance state to another, e.g., 11/7 to 8/5, which suggests a tilt or Zeeman-energy-induced-crossover between different FQHE states associated with different $\nu$. Further, we showed (see Fig. 2) that (i) “re-entrance” at $\nu =4/3$ (see Fig. 2a, c and e) includes also a shift in $R_{xy}/R_{K}$, (see Fig. 2b, d and f), (ii) an “avoided-crossing” type lineshape is suggested by the dotted lines in Fig. 2b, d and f around a 4/3 transition, (iii) there is also a size dependence in the angular interval associated with the $\nu = 4/3$ crossover (see Fig. 2a, c and e), (iv) there is a size dependence in the angle associated with the disappearance of the $\nu = 7/5$ at high tilt angles (Fig. 2a, c and e), and (v) there also appears to be a curvature in the track of the resistance minimum when plotted vs $R_{xy}/R_{K}$ for the 4/3, 7/5 and 11/7 (Fig. 2b, d and f). At the moment, it appears that the Hall resistance shift from $R_{xy}/R_{K} = (11/7)^{-1}$ to $R_{xy}/R_{K} = (8/5)^{-1}$, see Fig. 6, reflects a crossing-trajectory from one canonical FQHE to another. Perhaps, for $\nu \le 3/2$, curvature in the track of the resistance minima when plotted vs $R_{xy}/R_{K}$ reflect incomplete tilt induced crossovers, which require higher tilt angles and higher magnetic fields for their completion.

We begin by commenting upon the origin of size dependence in the angular interval in Fig. 2, marked by black horizontal line segments with colored arrows within them, for the disappearance of the “4/3” and “7/5” FQHE’s. A recent microwave power and temperature dependent study suggests that odd denominator rational fractional fillings of Landau levels, where the $R_{xx}$ minima disappear in a crossover range of tilt angles, exhibit so-called marginal metallic states, which are characterized by a profound temperature/microwave power insensitivity in the diagonal resistance^37^. In such a marginal metallic state, carrier, or composite fermion^29^, interaction with the boundary is likely to determine size effects. Note that the color plots suggest that the angular span for the absence of a resistance minimum depends on sample size. Characteristics size scales of relevance here could be the sample size (*W*), the carrier localization length, $l_{loc}$, and perhaps the phase coherence/inelastic lengths^38,39^. Assume that, in analogy to the metallic state observed at the center of Landau levels in the integral quantum Hall regime^38^, the carrier, or composite fermion^29^, localization length here is a function of the angle and varies similarly with angle in all three Hall devices. Further, for the sake of discussion, let us say that the localization length tends to diverge at some definite angle, say $\theta _{c} \sim 50^{0}$ for the “4/3” in Fig. 2a, c and e. As the localization length becomes larger on $\theta \rightarrow \theta _{c}$ from either side, the narrowest device $W = 100\,\mu m$ first satisfies the condition $W \ge l_{loc}$. Upon realizing this condition, carriers in this device are effectively delocalized as they interact with the boundary, this Hall bar becomes “metallic,” which leads to the vanishing of the resistance minimum and a temperature- or microwave power- independent diagonal resistance^37^. A closer approach to $\theta _{c}$ increases the localization length further such that the $W=200 \,\mu m$ specimen is the next to become “metallic,” followed by the $W=400 \,\mu m$ device with an even closer approach to $\theta _{c}$. Such an explanation invoking the reduced role for localization in the narrower sample provides a qualitative understanding for the size dependence of the angular width for the disappearance of the resistance minima for the 4/3 and 7/5 trajectories in Fig. 2^38^.

**Figure 7:**
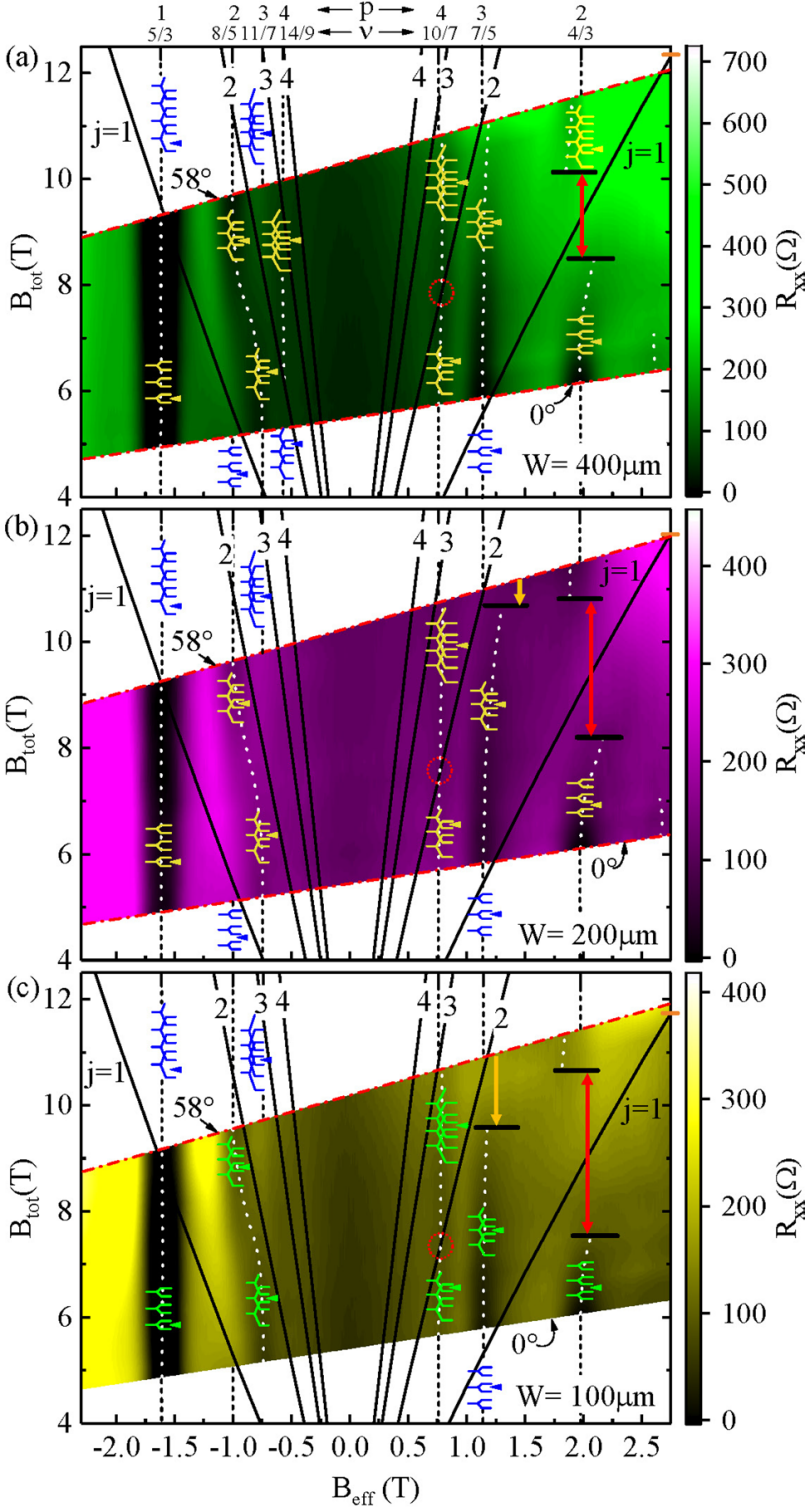
$R_{xx}$ color plots with coincidence fan charts versus $B_{eff}$ and $B_{tot}$. (**a**), (**b**) and (**c**) depict color plots of $R_{xx}$ versus $B_{tot}$ and $B_{eff}$ for *W*= 400, 200, and 100 $\mu m$, respectively. The dotted lines follow the $R_{xx}$ minima. The dashed lines mark specified odd-denominator fractional fillings of Landau levels. The black curved lines show the trajectory of the Zeeman-Landau level (LL) coincidence condition in the $B_{tot}$ - $B_{eff}$ space. The CF-LL occupancy in indicated by the Landau level cartoons. The horizontal lines in black, with the colored vertical arrowed lines in red and yellow, mark the boundary of the size-dependent angular interval where the diagonal resistance minimum vanishes. Here, $T=$ 55 mK.

Landau level crossings can occur upon varying the Zeeman energy with respect to the Landau level energies and such crossings can be manifested in transport^40,41^. Following the work of Du et al.^29^, which carried out such level crossing analysis in the FQHE regime, we provide here, for the sake of completeness, a composite-fermion-Landau-level (CF-LL) crossings analysis of the Fig. 2 data, see Fig. 7. In this approach, FQHE states around $\nu = 3/2$ are equivalent to IQHE states of composite fermions, of mass $m^*$ originating from $\nu = 3/2$, which are associated with CF Landau levels that are spaced by $\hbar eB_{eff}/m^*$ due to an effective magnetic field $B_{eff} = 3 (B_{\perp }- B_{\perp , 3/2})$ with $B_{\perp }$ and $B_{\perp ,3/2}$ the perpendicular component, and the perpendicular component at $\nu =3/2$, respectively, of the magnetic field. While $B_{eff}$ sets the field scale for CF-LL quantization, the Zeeman energy depends upon the total magnetic field $B_{tot} = B_{\perp }/cos (\theta )$. When the spin level of one CF Landau level coincides with the spin level of another CF Landau level due to, say, changing the tilt angle, the spectral gap disappears and $R_{xx}$ exhibits a relative resistance maximum. This coincidence condition is given by setting the Zeeman energy equal to integral multiples of CF-LL energy: $g^{*} \mu _{B} B_{tot} = j \hbar e B_{eff}/m^{*}$ with $j=1,2,3...$^29,33^. That is, at coincidence, the $B_{tot} = j B_{eff} (2m_0/(g^{*}m^{*})$, and the coincidence condition corresponds to lines in the $B_{tot}-B_{eff}$ space, whose slope depends on *j*^29^. In Fig. 7, we have displayed a color plot $R_{xx}$ versus $B_{tot}$ and $B_{eff}$ for the $W=400, 200,$ and $100\, \mu m$ sections. As in Fig. 2, two different line types are used here: dotted lines within the colored regions identify the trajectory of the resistance minima, while dashed lines in the white regions inside the graphs mark rational odd-denominator filling factors. The size dependence in the tilt angle intervals for the vanishing of the $\nu = 4/3$ and $\nu = 7/5$ resistance minima, previously seen in Fig. 2, are observable here (cf. Fig. 7a, b and c) over finite $B_{tot}$ intervals and marked with the vertical red and yellow arrowed lines, respectively. The center of the $B_{tot}$ interval where the 4/3 resistance minimum vanishes, for example, is equivalent to the point of maximum relative resistance utilized in the analysis by Du et al.^29^ Since this point of relative maximum resistance at 4/3 depends on device size, the fan charts and the associated parametric equation become dependent upon device size. Thus, $B_{tot} = j B_{eff} (2m_0/(g^{*}m^{*})$ with (a) $g^{*}m^{*}/2m_{0} = 0.190 + 0.012 (T^{-1}) B_{eff}$ for the $W=400 \,\mu m$ data, (b) $g^{*}m^{*}/2m_{0} = 0.195 + 0.012 T^{-1} B_{eff}$ for the $W=200 \,\mu m$ data, and (c) $g^{*}m^{*}/2m_{0} = 0.201 + 0.012 T^{-1} B_{eff}$ for the $W=100 \,\mu m$ data. In Fig. 7, a manifestation of the size dependence of the fan charts is the variable ordinate-intercept of the $j=1$ line at $B_{eff} = + 2.75T$, which is marked with a short horizontal orange-colored line. Notice that this intercept value decreases with decreasing *W*. For all three *W*, as mentioned, the $j=1$ line passes through the middle of the $B_{tot}$ interval where the 4/3 resistance minima vanishes. Similarly, the $j=2$ line passes through the estimated center of the $B_{tot}$ where the 7/5 resistance minima vanish. Notice also that the $j=2$ line crosses the dotted line marking the 10/7 resistance minimum. One might expect a vanishing of the 10/7 resistance minimum around the intersection of the $j=2$ line and the 10/7, at the small dotted red circle. However, this is not observed in the data. That is, a spin transition from one spin polarized 10/7 state to another is not observed in these data although it is suggested by the fan chart. Over the span $B_{eff} < 0$, the observable 14/9 resistance minimum falls between the $j=2$ and $j=3$ lines of coincidence for $W=400\, \mu m$. Notice that the 14/9 minimum trajectory is only observable in Fig. 7a, and not in 7b or c. The 11/7 to 8/5 crossover, which is observable in panels Fig. 7a–c, remains to be understood in the context of this CF-LL crossing plot. We remark, however, that the slope of the dotted line indicating the crossover is approximately the same as the slope of the neighboring $j=2$ line. Du et al.^29^ have used such level crossing analysis to determine the $B_{eff}$-dependence of $g^{*}$ and $m^*/m$. Although a detailed analysis is beyond the scope of this work, we remark that, for $W=400\, \mu m$, the results ($g^{*}m^{*}/2m_{0} = 0.190 + 0.012 (T^{-1}) B_{eff}$) are also approximately consistent with $m^{*}/m = 0.65 + 0.00158 B_{eff}$ and $g^{*} = 0.57 + 0.035 B_{eff}$. This expression for $g^{*}$ suggests a value $g^{*} =0.43$ at $\nu = 2$ and $g^{*} = 0.88$ at $\nu = 1$, which are consistent with expectations for observing a bare g-factor at $\nu =2$, and an exchange enhanced value at $\nu = 1$.

So far as the observation of a size dependence of $g^{*}m^{*}$ is concerned, our result, that (a) $g^{*}m^{*}/2m_{0} = 0.190 + 0.012 (T^{-1}) B_{eff}$ for the $W=400 \,\mu m$ data, (b) $g^{*}m^{*}/2m_{0} = 0.195 + 0.012 T^{-1} B_{eff}$ for the $W=200 \,\mu m$ data, and (c) $g^{*}m^{*}/2m_{0} = 0.201 + 0.012 T^{-1} B_{eff}$ for the $W=100 \,\mu m$ data, represents a set of three parallel lines with the same slope ($0.012 T^{-1}$) in a plot of $g^{*}m^{*}/2m_{0}$ versus $B_{eff}$. The question arises whether the observed size dependence should be attributed to a size dependent $g^{*}$, or $m^{*}$, or both. Consider first the g*: The exchange enhanced spin gap can be written as: $\Delta _{spin} = g^{*} \mu _{B} B = g_{0} \mu _{B} B + E_{Ex}$, where $g^{*}$ ($g_{0}$) is the enhanced (bare) g-factor^42^. If overlapping Landau levels may be neglected, and at sufficiently low temperature, as in our experiments, one might simply write $E_{Ex} = \Sigma (n^{-} - n^{+})$, where $\Sigma$ is a self energy, and $n^{-}$ ($n^{+}$) are the concentrations in the two spin subbands^43^. Although the self energy $\Sigma$ can potentially be size dependent if the separation of an electron-hole pair becomes limited by the sample size^42^, the exchange enhancement of the g-factor ought to vanish due to the concentration difference dependence of the exchange term, when the two spin subbands of the lowest Landau level are equally occupied as at $\nu = 2$. That is, one naively expects a size independent g-factor at $\nu =2$. Yet, the results suggest a size dependent $g^{*}m^{*}$ even at $\nu =2$. Due to this feature, and the simple parallel line behavior of $g^{*}m^{*}$ for the three sections, we suggest that the size dependence of $g^{*} m^{*}$ possibly originates from a size dependence to $m^{*}$ only, with a larger $m^{*}$ in the smaller section of the sample. Since composite fermion masses are known to diverge as the filling factor approaches $\nu =1/2$ and scale as $n^{1/2}$^44^, perhaps it is plausible that it could also depend on the specimen size.

Finally, the tilt induced transformation of the 11/7 to the 8/5 with increasing angle (Fig. 3a, b) coincides with a decreasing activation energy for the 11/7 followed by an increasing activation energy for the 8/5 with increasing tilt angle, see Fig. 4a. In reference to Fig. 7, this feature suggests that the mobility gap at $p = 3$ (11/7) collapses as the mobility gap at $p = 2$ (8/5) becomes larger with increasing tilt angle. From the CF-LL scheme exhibited in Fig. 7, the $p=2$ (8/5) crossover from the unpolarized to polarized spin state over the corresponding $j=1$ CF-LL line, see Fig. 7, occurs close to $\theta = 0^{0}$. This suggests a possible collapsed gap at the outset, followed by a progressively stronger polarized spin state with an increasing mobility gap for the 8/5 for the entire range of experimentally accessible tilt angles. The CF-LL scheme exhibited in Fig. 7 also suggests that $p=3$ (11/7) corresponds to a partially polarized state at the outset, with a trajectory towards a crossing of the $j=2$ CF-LL Line at the highest experimentally accessible angle, implying a decreasing activation energy with increasing angle. So, the observed trends in the activation energies, see Fig. 4a, are not inconsistent with Fig. 7, although some expected crossovers (dotted red circles in Fig. 7) are not manifested. The surprising feature here is, however, that one fractional state (11/7) is extinguised in favor of another (8/5) with increasing tilt. Since the associated filling factors are so close to each other, and resistance minima have a finite width in filling factor, it could be that, due to overlap and proximity, the stronger fraction simply competes against and consumes the weaker one, per experimental observation (see also the Supplementary Material).

## Conclusions

In summary, these results show that tuning the spin energy by tilting the specimen can produce fractional quantized Hall effect transformations that include both a change in $\nu$ for the $R_{xx}$ minimum, e.g., from the $\nu = 11/7$ to the $\nu = 8/5$, and a change in the $R_{xy}$, e.g., from $R_{xy}/R_{K} = (11/7)^{-1}$ to $R_{xy}/R_{K} = (8/5)^{-1}$, with increasing tilt angle. Further, the results showed a striking size dependence in the tilt angle interval for the vanishing of the 4/3 and 7/5 states, and concurrent observable shifts of $R_{xy}$ at the $R_{xx}$ minima- the latter occurring in the vicinity of $\nu = 4/3, 7/5$ and the 10/7, see Fig. 2b, d and f. The results demonstrate both size dependence in the FQHE regime and the possibility, not just of competition between different spin polarized states at the same $\nu$ and $R_{xy}$, but also the tilt or Zeeman-energy-dependent-crossover between distinct and different FQHE.

## Methods

The GaAs/AlGaAs heterostructures used in these studies were characterized by a sheet electron density $n_{0} (55mK) = 2 \times 10^{11}\, cm^{-2}$ and an electron mobility $\mu (55\,mK) = 1.4 \times 10^{7} \,cm^2/Vs$ after brief illumination during cooldown^45^. Hall bars^46–49^ were fabricated by standard photolithography from the MBE grown single interface structure material including a triangular quantum well. The thickness of the 2D electron system is estimated to be ca. 50 nm. Examined Hall devices included sections with widths $W = 400, 200,$ and $100\, \mu m$ as the length-to-width ratio $L/W = 1$. Similar specimens were examined in other size dependence studies^31^. Electrical contacts were formed by depositing and alloying Au-Ge/Ni at the Hall bar contact pads. The sample was wired into a chip carrier, loaded into a dilution refrigerator system, with the sample situated at the center of a superconducting solenoid, and the electrical response was measured using low frequency lock-in based techniques. The applied current to the sample, *I*, was measured together with the diagonal and Hall voltages. The diagonal and Hall resistances were calculated as the voltages divided by the current. The sample could be tilted in-situ using a geared mechanical system; the tilt angle was determined from the expected $B_{\perp }$ dependence of the Hall effect and a supplementary angular sensor. Magnetic field sweeps were carried out at fixed increments of $\cos (\theta )$ for the color plots shown in Figs. 2, 3, 5 and 7. Activation energies were measured also using the techniques discussed in ref.^37^

## Supplementary Information

Supplementary Information.

## Data Availability

The datasets generated during and/or analysed during the current study are available from the corresponding author on reasonable request.
