# Supplementary material for: Size dependence- and induced transformations- of fractional quantum Hall effects under tilted magnetic fields
Source: Sci Rep. 2022 Nov 10;12:19204. doi: 10.1038/s41598-022-22812-x (PMC9649807; doi:10.1038/s41598-022-22812-x)
Supplement: Supplementary file 1 — Supplementary Information. [file 41598_2022_22812_MOESM1_ESM.pdf]

Supplementary Information

The GaA/AlGaAs heterostructure devices examined for the study above exhibited also a low density ($n \sim 1.2 \times 10^{11} cm^{-2}$), low mobility ($\mu \sim 6.6 \times 10^{6} cm^{2}/Vs$) condition, when the specimens were cooled in the dark. These specimens, in this low mobility condition, exhibited fewer perceptible FQHE over the interval $1 \leq \nu \leq 2$. Yet, the $5/3$ and $4/3$ FQHE's were prominent in the untilted ($\theta = 0^{0}$) condition, with the specimen normal parallel to the magnetic field, see supplementary figure 1(a). At a tilt angle of $\theta = 60^{0}$ of the specimen normal with respect to the magnetic field, see supplementary figure 1(b), the $5/3$ minimum becomes deeper, as the $4/3$ disappears. In comparing panels (a) and (b), we observe that the $\nu = 4/3$ resistance minimum of panel (a) becomes unobservable in panel (b), while an imperceptible $\nu = 7/5$ resistance minimum in panel (a) becomes observable in panel (b).

Color plots of R_{xx} with $\cos(\theta)$ as the ordinate, and with ν as the abscissa, are shown in supplementary figure 2 (a), (c), and (e). Dark bands in the figure indicate resistance minima and the trajectory of the minima are indicated by the dotted lines; short white vertical lines within the figure indicate ν associated with observable FQHE in the exhibited ν interval. In supplementary fig. 2(a), (c), and (e), the $5/3$ FQHE minimum is prominent, it runs mostly vertically, and bends towards lower filling factors at the highest angles. The $4/3$ is observable over the approximate angular span $0 \leq \theta \leq 45^{0}$. Once the $4/3$ minima disappear, the $7/5$ minima become observable and these minima appear to bend towards higher filling factors with increasing angle. The $7/5$ minima become unobservable above $\theta \approx 63^{0}$. An obvious size dependence is not discernable in

this low density, low mobility condition.

Color plots of R_{xx}, with R_{xy}/R_K as the abscissa, and $cos(\theta)$ as the ordinate are exhibited in supplementary figure 2(b), (d), and (f). Supplementary figure 2(b), (d), and (f) show some of the same general features as supplementary figure 2(a), (c), and (e) including these differences: (i) The dark bands associated with the R_{xx} minima appear narrower here, as also observed in the high mobility condition. (ii) An appreciable shift is observable in the Hall resistance of the $5/3$ at the highest angles, $\theta \geq 60^0$. This feature seems to suggest that the $5/3$ could disappear at even higher angles. (ii) the dark band associated with the $4/3$ resistance minimum tracks to higher values of R_{xy}/R_K with increasing angle before it vanishes. (iii) the dark band associated with the $7/5$ resistance minimum tracks to smaller values of R_{xy}/R_K before it too vanishes. In the vicinity of the crossover point where the $4/3$ vanishes and the $7/5$ appears at around $\theta = 49^0$, the lineshapes of two branches together are reminiscent of an "avoided crossing".

Supplementary figure 3 shows plots of R_{xx} vs. $\cos(\theta)$ along resistance minima (dotted lines) in supplementary figure 2 for $W = 400\mu m, 200\mu m,$ and $100\mu m$ wide sections in panels (a), (b), and (c), respectively. All three panels show that the $5/3$ resistance drops with increasing angle to $\theta \approx 60^0$ before increasing again at higher angles. We note that the increase in R_{xx} for $\theta \geq 60^0$ correlates with the shift of the corresponding dark bands away from $R_{xy}/R_K = (5/3)^{-1}$ in supplementary figures 2(b),(d), and (f). In addition, the $4/3$ resistance increases with increasing θ (or, equivalently, decreasing $\cos(\theta)$). It looks like the $7/5$ becomes visible when its resistance falls below the $4/3$ resistance.

Supplementary figure 4 shows the measured R_{xy}/R_K vs. $\cos(\theta)$ along the dotted lines shown in supplementary figure 2. Here, systematic shifts are observable in R_{xy}/R_K for the $4/3$, $7/5$, and $5/3$ as a function of $\cos(\theta)$.

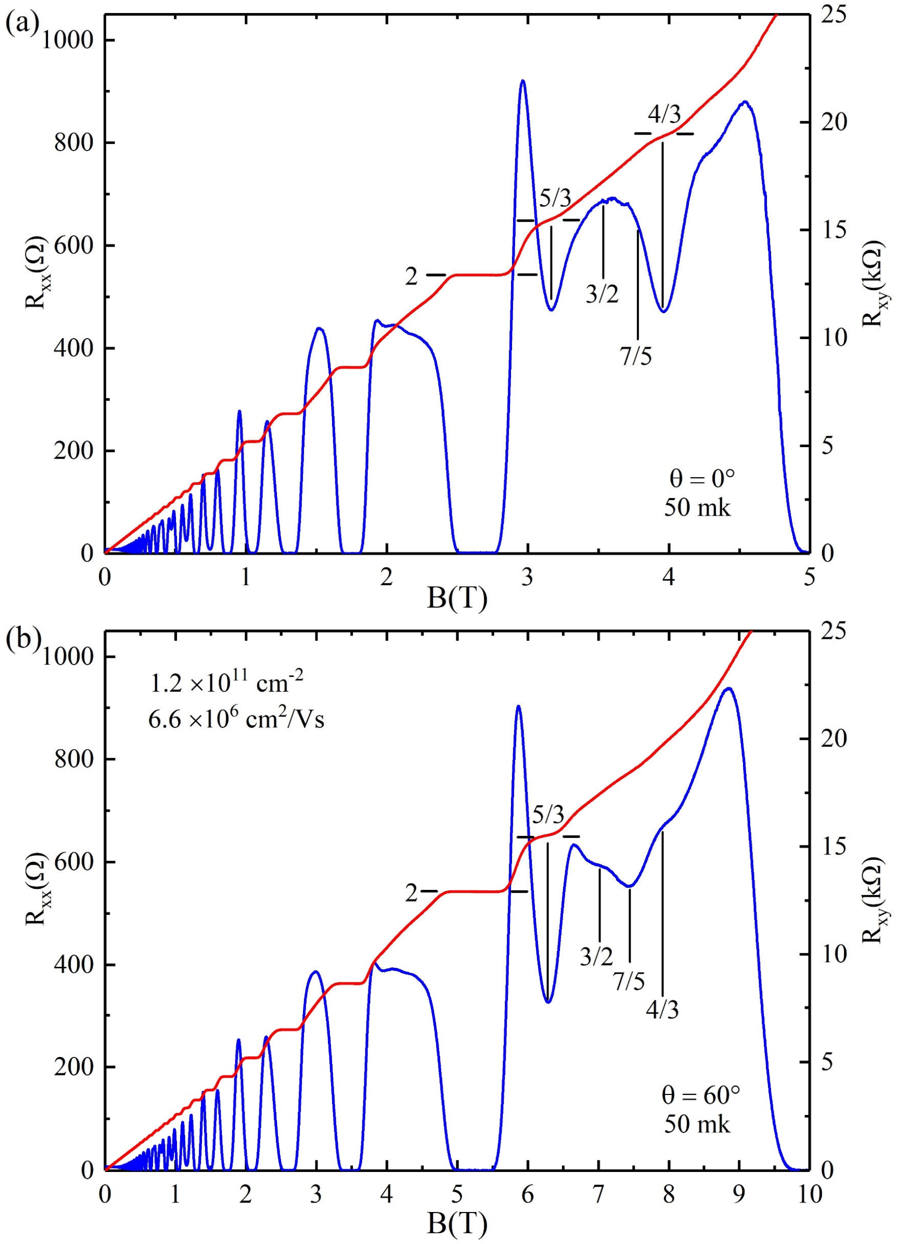

Supplementary Figure 1 : **The diagonal and Hall resistances versus the magnetic field in a GaAs/AlGaAs heterostructure in the low mobility, low density condition.** The diagonal resistance (R_{xx}) and Hall resistance (R_{xy}) are shown with some marked Integral (I) and Fractional (F) Quantized Hall Effects (QHE). (a) The R_{xx}- and R_{xy}- vs B at a tilt angle $\theta = 0^0$, where the sample normal is parallel to the magnetic field, i.e., $\theta = 0^0$. (b) The results at a tilt angle $\theta = 60^0$.

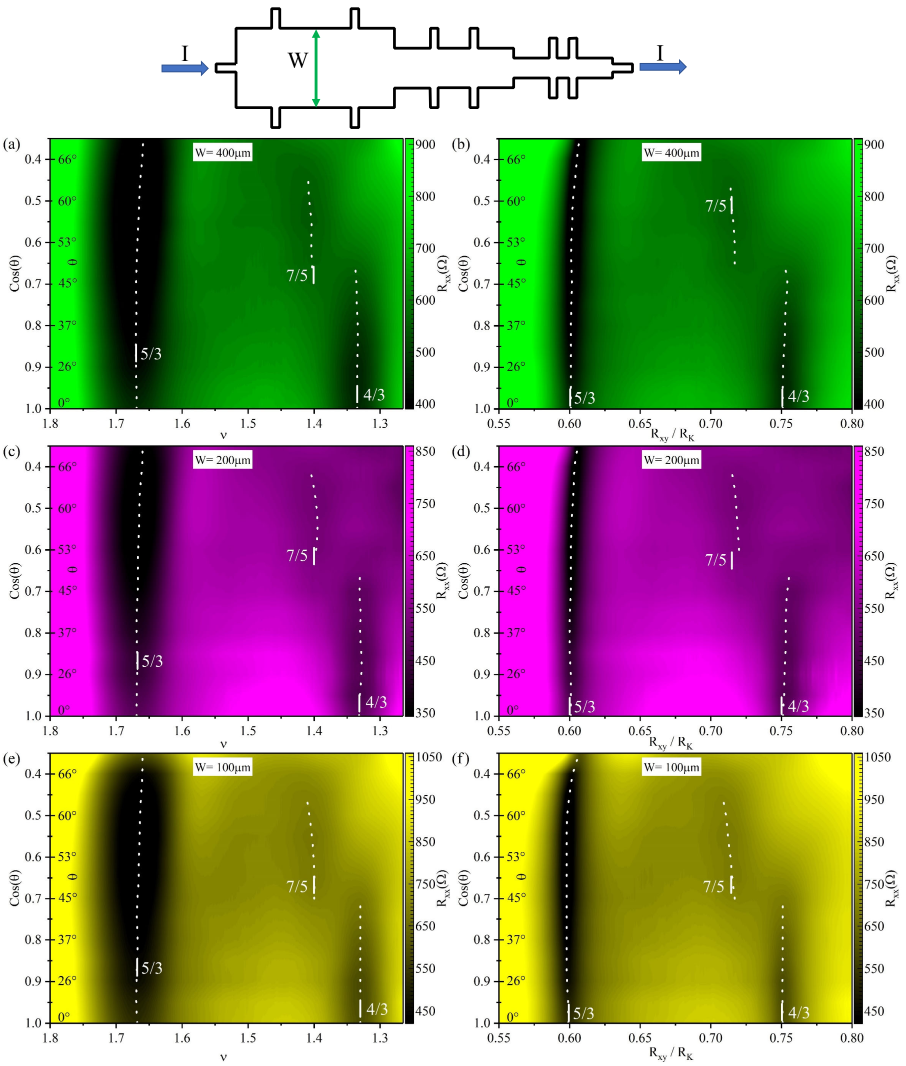

Supplementary Figure2 : **Color plots of the tilt field effect in a low density specimen with different widths (W).** (Top): The Hall bar geometry including three different widths, W, with length (L) to W ratio for the contacted regions L/W = 1. (a), (c), and (e) depict color plots of R_{xx} vs. cos(θ) and vs. ν for W= 400, 200, and 100 μm, respectively. (b), (d), and (f) shows color plots of R_{xx} vs. cos(θ) and vs. R_{xy}/R_K for W= 400, 200, and 100 μm, respectively. The dotted lines follow the R_{xx} minima. Here, $T = 55$ mK, and $n = 1.2 \times 10^{11} cm^{-2}$.

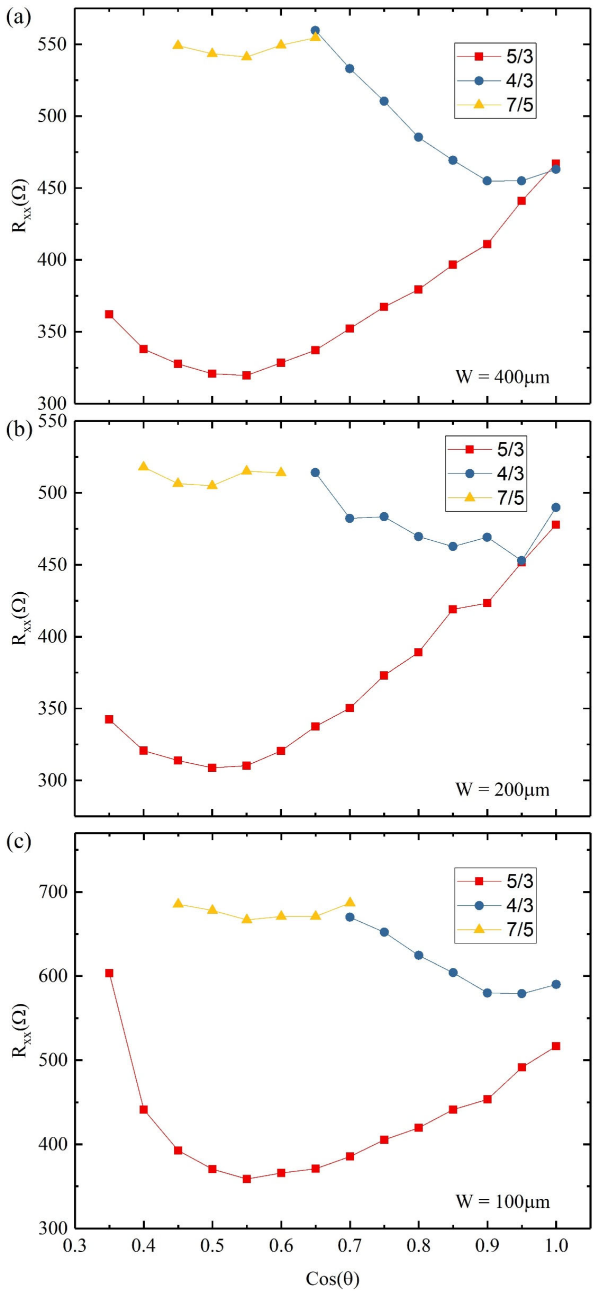

Supplementary Figure 3 : R_{xx} **is plotted versus** $cos(\theta)$ **along the dotted lines shown in the previous figure.** The minima diagonal resistance (R_{xx}) is shown vs. $cos(\theta)$ in panels (a), (b), and (c), for the $W = 400\mu m, 200\mu m,$ and $100\mu m$ sections, respectively.

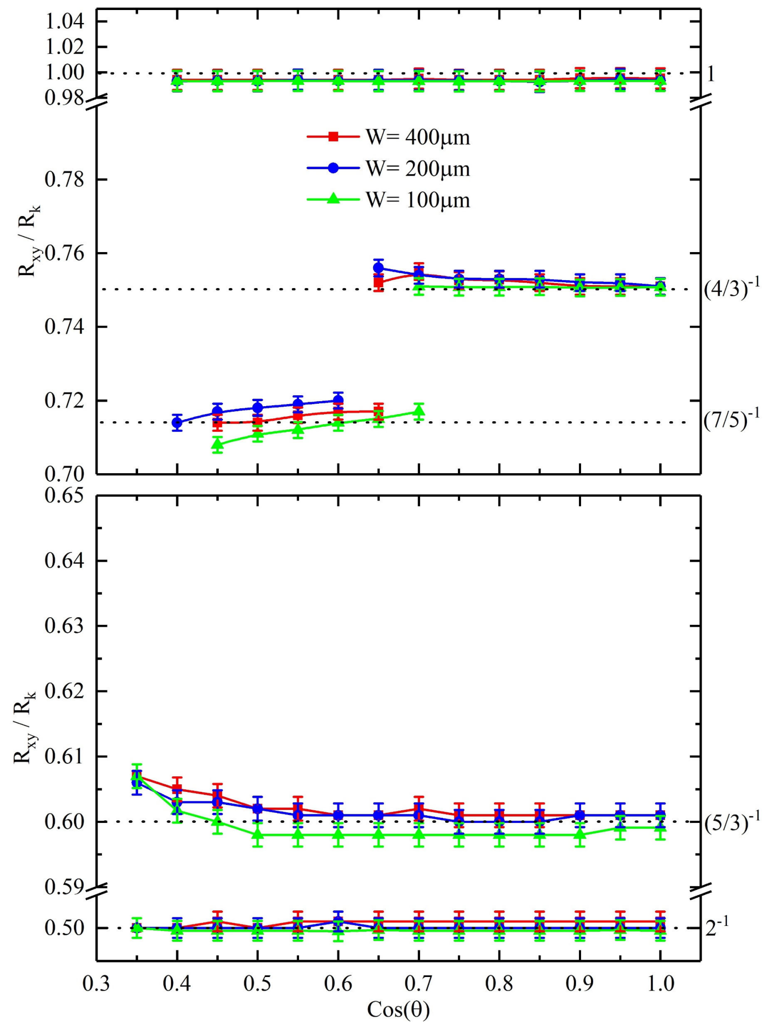

Supplementary Figure 4 : **Measured R_{xy}/R_K values at the diagonal resistance minima vs. cos(θ) in Hall bars with $W = 400, 200,$ and $100\mu m$.** This figure illustrates the observed R_{xy}/R_K vs. $\cos(\theta)$, where θ is the tilt angle, at the corresponding R_{xx} minima for the sample widths of 400, 200, and 100 micrometers. The dotted lines indicate the expected R_{xy}/R_K values at the well known fractional states labeled on the right ordinate. Note that, at $\nu = p/q$, where p/q is a rational fraction, one expects Hall resistance $R_{xy}/R_K = (p/q)^{-1}$ The top panel examines $R_{xy}/R_K > (3/2)^{-1}$ while the bottom panel examines $R_{xy}/R_K < 3/2^{-1}$

.
